# Factors associated with alcohol use among minority female adolescents receiving preventive text messaging for underage drinking

**DOI:** 10.1186/1940-0640-10-S2-P10

**Published:** 2015-09-24

**Authors:** Meenal Sawant, MM Hospital, EF Wagner, SL Morris, LM Siqueira

**Affiliations:** 1 School of Social Work, Florida International University, Miami, USA; 2 Adolescent Medicine Unit, Nicklaus Children's Hospital, Miami, USA

## Background

There is a high prevalence of alcohol use among adolescents, especially among Hispanic teens in middle adolescence. This emphasizes the need for developmentally appropriate and culturally sensitive underage drinking brief interventions. Texting (i.e. SMS) is extremely popular among U.S. teenagers, and its advantages as a brief intervention includes wide reach, low cost, easy standardization, automation of health message delivery, and the ability to include multiple recipients concurrently.

## Material and methods

We conducted an RCT to examine the effectiveness of preventive text messaging for underage drinking among a sample of predominantly (77.0%) Hispanic youth. Participants (n = 375) were recruited from a large, urban adolescent medicine setting. After completing the baseline intake survey, each participant was randomly assigned to either intervention (i.e. 2 times/week alcohol-related prevention SMS's for 16 weeks) or control (assessment-only) groups. Follow-up assessments were conducted for all participants at post-treatment and 1-month post-treatment. In the present study, we analyzed baseline data to examine the association between psychosocial risk factors and past month alcohol use.

## Results

The sample included females between 12 and 18 years old (M= 15.90, SD= 1.49). 42% reported lifetime alcohol use and 24% reported past month use. Regression analyses (MPLUS; version 5) revealed that past month alcohol use and future drinking intentions were significantly associated with (a) alcohol availability (ß = 0.315 and ß = 0.115 respectively), and (b) the CRAFFT (screening tool for substance use risks and consequences; ß = 1.312, ß = 0.174 respectively).

## Conclusions

These findings highlight the importance of considering factors such as availability of substances and drinking intentions in the development of underage drinking brief interventions for minority populations.

